# The virtual supermarket: An innovative research tool to study consumer food purchasing behaviour

**DOI:** 10.1186/1471-2458-11-589

**Published:** 2011-07-25

**Authors:** Wilma E Waterlander, Michael Scarpa, Daisy Lentz, Ingrid HM Steenhuis

**Affiliations:** 1 Department of Health Sciences and EMGO+ Institute for Health and Care Research, VU University, De Boelelaan 1085, 1081 HV Amsterdam, the Netherlands; 2 Aardman Studios, Gas Ferry Road, Bristol, BS1 6UN, UK; 3 Department of Health Sciences and EMGO+ Institute for Health and Care Research, VU University, De Boelelaan 1085, 1081 HV Amsterdam, the Netherlands; 4 Department of Health Sciences and EMGO+ Institute for Health and Care Research, VU University, De Boelelaan 1085, 1081 HV Amsterdam, the Netherlands

## Abstract

**Background:**

Economic interventions in the food environment are expected to effectively promote healthier food choices. However, before introducing them on a large scale, it is important to gain insight into the effectiveness of economic interventions and peoples' genuine reactions to price changes. Nonetheless, because of complex implementation issues, studies on price interventions are virtually non-existent. This is especially true for experiments undertaken in a retail setting. We have developed a research tool to study the effects of retail price interventions in a virtual-reality setting: the Virtual Supermarket. This paper aims to inform researchers about the features and utilization of this new software application.

**Results:**

The Virtual Supermarket is a Dutch-developed three-dimensional software application in which study participants can shop in a manner comparable to a real supermarket. The tool can be used to study several food pricing and labelling strategies. The application base can be used to build future extensions and could be translated into, for example, an English-language version. The Virtual Supermarket contains a front-end which is seen by the participants, and a back-end that enables researchers to easily manipulate research conditions. The application keeps track of time spent shopping, number of products purchased, shopping budget, total expenditures and answers on configurable questionnaires. All data is digitally stored and automatically sent to a web server. A pilot study among Dutch consumers (n = 66) revealed that the application accurately collected and stored all data. Results from participant feedback revealed that 83% of the respondents considered the Virtual Supermarket easy to understand and 79% found that their virtual grocery purchases resembled their regular groceries.

**Conclusions:**

The Virtual Supermarket is an innovative research tool with a great potential to assist in gaining insight into food purchasing behaviour. The application can be obtained via an URL and is freely available for academic use. The unique features of the tool include the fact that it enables researchers to easily modify research conditions and in this way study different types of interventions in a retail environment without a complex implementation process. Finally, it also maintains researcher independence and avoids conflicts of interest that may arise from industry collaboration.

## Background

The prevalence of overweight and obesity is rising worldwide, with the largest burden among groups with a lower socio-economic status [1-3]. These figures are worrying since overweight and obesity are related to numerous chronic diseases, including type 2 diabetes mellitus, several types of cancer, and cardio vascular diseases [4]. Much research has been conducted in the search for effective interventions which will decrease the prevalence of overweight and obesity. Whereas most of these interventions have been targeted at individual determinants of dietary intake, today it is increasingly recognized that environmental factors also contribute to obesity and that environmental interventions should also be considered [5].

According to the ANGELO-Framework (Analysis Grid for Environments Linked to Obesity), economic factors are one of the influential environmental factors in food choice [5]. Since there is a large body of evidence showing that currently the healthier choice is the more expensive choice [6,7], pricing strategies seem a promising intervention to change dietary behaviour, especially for groups with a lower socio-economic status. Both marketing and consumer research have indicated that price is one of the most important tools in steering consumer behaviour [8,9]. Nonetheless, to date little is known on what the most effective pricing strategy would be. In a previously conducted Delphi Study we examined expert standpoints on what type of pricing strategies would be most feasible and effective in stimulating healthier dietary behaviours. The expert panel agreed on the potential success of offering small gifts, offering price-cuts on healthy foods and discounting healthier foods more frequently [10]. A systematic review of the effectiveness of economic incentives in changing dietary behaviour showed promising results for such strategies indeed [11]. Nevertheless, this review also concluded that the evidence is limited and restricted to small-scaled interventions with a short running period. Examples of such interventions include studies using vending machines or school canteens [12,13].

While the potential effects of pricing strategies to stimulate healthier food choices are promising, before pricing strategies are introduced on a large scale, information on the effectiveness of such strategies in a broader setting where people obtain most of their groceries is required, that is a retail environment. To our knowledge, the only example of a large-scaled randomized controlled trial on the effects of retail pricing strategies is the New Zealand SHOP study. This study evaluated the effects price reductions of healthier foods (12.5%) and nutrition education on supermarket purchases among 1,104 consumers. The authors found that the pricing intervention alone lead to more healthy food purchases [14].

As SHOP is the only supermarket trial on food pricing strategies available at present, more research is needed to learn about the actual effects of food pricing strategies. These studies are important complementing those that use data from large datasets (e.g., household expenditure surveys) and thereby gaining insight into complex processes such as 'own-price elasticity' (the change in demand for a product as a consequence of a change in price of that product) and 'cross-price elasticity' (the change in demand for a product in response to a change in the price of another product) [15]. However, such studies are complex, mostly with regard to implementation issues. In order to find a solution to this problem, we developed a research tool which can be used to study the effects of price interventions in a virtual-reality setting: the Virtual Supermarket. This software tool can be used to study various pricing interventions in a supermarket setting without having to rely on a complex implementation process. Below, the development and implementation of this software application will be explained and the results of a pilot study on the effectiveness of the application will be presented.

### Implementation

**Figure 1:**
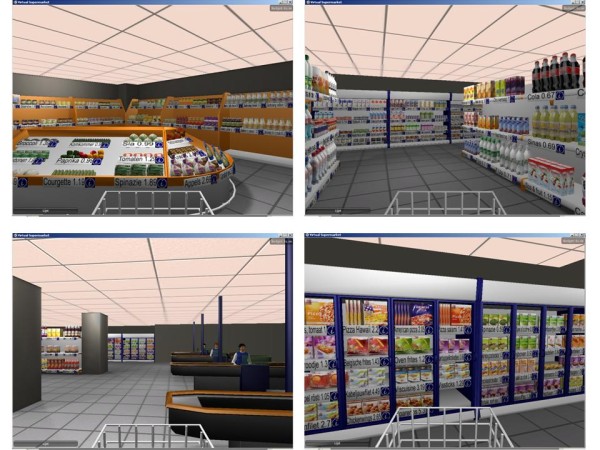
**Screenshots of the Virtual Supermarket**.

In this section we will describe the development, design and capabilities of the Virtual Supermarket. The Virtual Supermarket is a three-dimensional software application (Figure 1) with which study participants can shop in a manner comparable to a real supermarket. The application was developed in collaboration with SARA Computing and Networking Services Amsterdam^a^. The Virtual Supermarket contains a front-end which can be seen by the participants and a back-end that enables researchers to easily manipulate research conditions. The application can be obtained via an URL and the installation consists of unpacking a compressed file at the desired location. The application is available for both MS Windows (Windows 2000+) and Mac (OS10+) and is built to accommodate a wide range of computer arrangements, by allowing the user to choose different screen sizes and graphical quality. Researchers can download the application for free and in a way that preserves their anonymity. More details about obtaining the software can be found under the subheading 'Availability and requirements' at the end of this paper.

#### The front-end of the Virtual Supermarket

**Figure 2:**
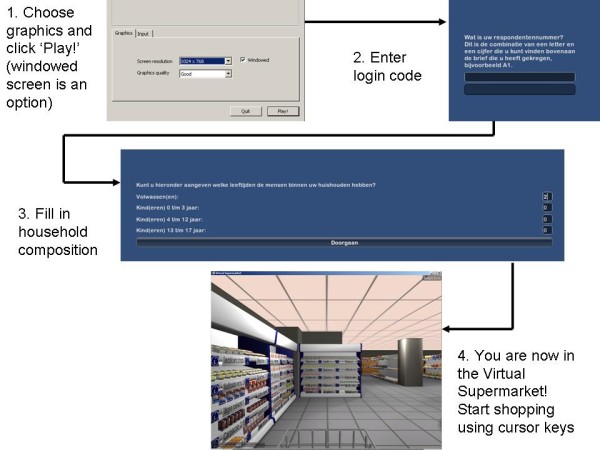
**Procedure for starting shopping in the Virtual Supermarket**.

The procedure for starting to shop in the Virtual Supermarket is shown in Figure 2. The Virtual Supermarket was designed in the image of a real supermarket using an Amsterdam branch of the Dutch market leader as a model. Photographs of real products were used to compose products for the Virtual Supermarket and prices were made available through shelf labelling. Food prices were based on the prices of the two Dutch market leaders, and the stock was also based on a real supermarket. For this, we used figures provided by one of the major Dutch supermarket specialist journals and information from the market leader's website [16]. It was found that an average Dutch supermarket offers about 7,000 different food products. Since this number contains approximately 200 different types of cheese and 250 varieties of wine, it was decided that a representative product selection should be made using the 38 different food categories on the market leader's website. These categories comprise, for example, potatoes, vegetables, poultry, fish, soft drinks, confectionary, and bread [16]. Within each product category, a sample representing approximately 10% of the usual stock was selected by choosing popular and frequently consumed products. Due to lack of sales data, the identification of popular products was conducted by the authors (WW and IS). The actual total number of products was greater than 10% because different types within products were counted as one. Products in the Virtual Supermarket could represent a number of product varieties such as for example grapes represented red and white grapes and fruit yoghurt represented peach/strawberry/and forest fruit flavours. The program mentions this to the participants as they move their mouse to a product. Furthermore, to ensure the availability of both healthy and unhealthy options, both products that met and products that didn't meet nutritional guidelines were chosen within each product category. For this purpose we used nutrition profiling criteria from the Choices front-of-pack nutrition logo. These criteria are based on the international WHO recommendations regarding saturated fatty acids, trans fatty acids, sodium, and added sugar [17]. Finally, for most products no different package sizes were available, only regular package sizes were included. Also, no specific brands were included in the stock of the Virtual Supermarket. For each product, only one type is available, without linkage to a specific brand name, although for some products we did include both the regular and diet options, and we also distinguished between whole and refined grain variations. As a consequence, the product appearance in the Virtual Supermarket was somewhat different to that of a real supermarket, but it enabled us to select a wider product range (e.g., cola, orangeade and water rather than Coca-Cola, Pepsi Cola and own-brand cola) within each product category. In addition, it prevented the interaction of branding effects with other effects (such as pricing).

**Figure 3:**
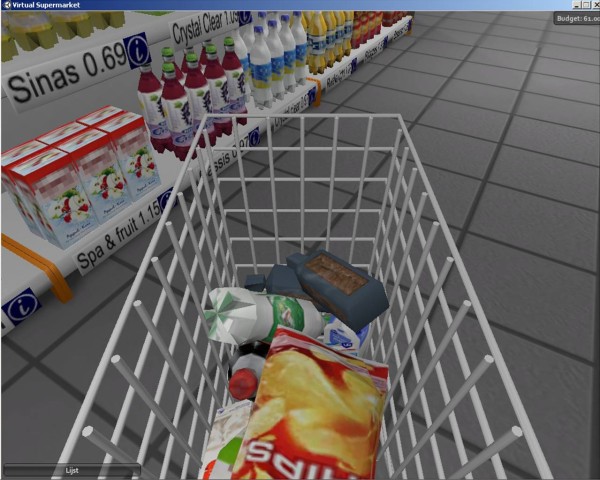
**Shopping cart in the Virtual Supermarket**.

Product purchasing in the Virtual Supermarket was also designed to be comparable to purchasing in real life. The application allows participants to read the nutritional label on the products by clicking on an information symbol next to the product. Consumers can select groceries by clicking on them and the product then appears in their shopping cart (Figure 3). While shopping, a list of selected groceries is visible, including the price and total amount of money spent thus far. Participants can use this list to change products or delete them altogether from their shopping cart. The application also has a feature which calculates a specific budget for shopping. This amount is calculated on the basis of the data from the Dutch National Institute of Budget Education (NIBUD), which provides average food expenditure data for different household sizes and compositions. Participants are asked about their household composition and are allocated a corresponding food budget. This budget was done because it forces people to make price sensitive choices. However, if researchers wish to use different types of budget allocation this can easily be achieved.

After collecting all of their groceries in their shopping cart, consumers in the Virtual Supermarket go to the checkout and, if they have not exceeded their designated budget the application proceeds. If the budget is exceeded, participants are asked to remove some of their groceries. After finishing the shopping task, it is possible to include several configurable questionnaires which the participant must complete before the application transmits the data and closes.

#### The back-end of the Virtual Supermarket

**Figure 4:**
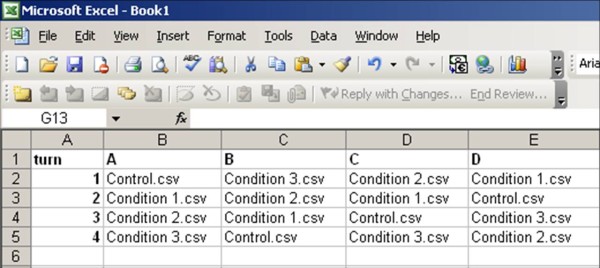
**Impression of a CSV file determining specific conditions attached to specific letters of the alphabet and the number of turns by a participant as seen in the back-end of the Virtual Supermarket**.

The back-end of the Virtual Supermarket is designed in such a way that it can be used by researchers to change research conditions in the application without the assistance of an expert programmer. Researchers can use the back-end to change the budget calculation, food prices, food labels, the placement of signs, and to configure questionnaires. The back-end can also be used to create different research conditions. For example, research condition A might be linked to regular prices and research condition B to discounted prices. Participants who log in to the application with login code A (1-1000) will see regular prices and participants who log on with login code B (1-1000) will receive discounted prices (Figure 4). Every letter of the alphabet can be assigned to different research conditions, so in theory one could run an experiment with 26 different conditions. Along with the different research conditions, the application also allows one participant numerous turns. Thus, one participant can shop under different conditions (Figure 4). All of these changes can be made using comma-separated values in Excel or using a text editor.

#### Data processing

A key feature of the Virtual Supermarket is that it digitally collects and stores data. The application keeps track of all products purchased by a participant, the time at which the products were selected, the allocated budget, total expenditures and answers to configurable questionnaires. When a respondent completes the virtual shopping task, data is automatically sent to a server and stored in a unique comma-separated value file under the respondent's personal code. The server constantly measures whether it has collected all files. All collected single respondent files can be imported into one Excel data file using a specifically developed web service.

#### Using the Virtual Supermarket

If researchers are interested in using the Virtual Supermarket for their own research, they can use the information provided in this manuscript and on the projects homepage to find out how the program works. Furthermore, they can download the application to their computer and have a close look into the software. Furthermore, we are also happy to provide the Virtual Supermarket application to other researchers so that they can use it in their own research. The tool is ready to use for Dutch food pricing experiments, but the application was designed in such a way that it can also be used for different types of experiments, such as for example on product placement or supermarket lay out. If researchers are interested, we can provide the software basis, from which they can develop their own Virtual Supermarket. More detailed information about configuration and use of the Virtual Supermarket can be found at the projects' homepage: http://www.bioinformatics.org/supermarket/index.html

## Results

**Figure 5:**
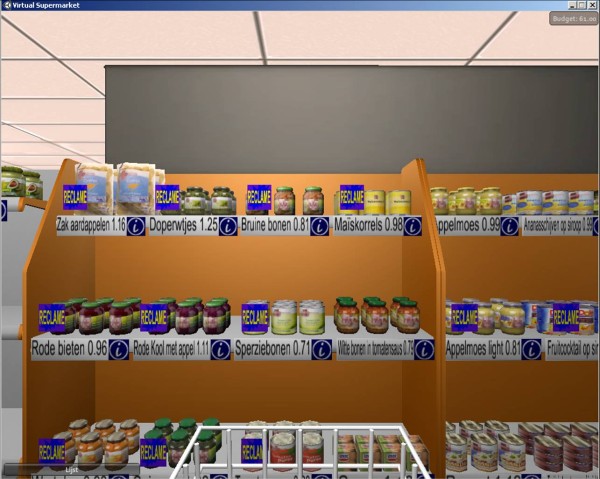
**Sales signs in the Virtual Supermarket**.

The Virtual Supermarket can be used to study several food pricing strategies in a supermarket environment. It can also be used to study the effects of signage or labelling products, for example by adding sale signs or healthy food labels (Figure 5). To date, the application has been successfully used in five experiments among 557 participants. These experiments studied the effects of several discount percentages on the purchase of healthy food products, the effects of combining discounts on healthier food products with price increases on unhealthy products, the effects of different signs ('healthy choice', 'sale' and 'sale & healthy choice'), and the effects of a tax measure on sugar-sweetened beverages. The Virtual Supermarket was first tested among 38 university staff members. Subsequently, to gain a better insight into its accuracy, we conducted a pilot study among consumers. The aim of this pilot was to test the workability and accurateness of the Virtual Supermarket and to examine how people judge the program. The methods and results of this pilot study are described below.

### Pilot study

#### Methods

Participants were recruited through local and national newspapers, the Amsterdam public library, and community centers in Amsterdam. The inclusion criteria were that people had to be 18 years of age or older, be familiar with the Dutch language, and run their own household. Participants were asked to complete a weekly grocery shop for their household at the Virtual Supermarket. They received a personal code which gave them access to the Virtual Supermarket and written instructions about the program. Most participants completed the experiment at home. At the start of the study, participants were asked a few background questions about characteristics such as gender, ethnicity, household composition, degree of responsibility for the grocery shopping, weekly food budget, education level, employment status, and household income. Unfortunately, data on age was not available. However, because it may be expected that younger participants would find it easier to work with a computer tool, the study sample merely included middle-aged participants. After finishing shopping, participants were asked to respond to eight items concerning the use of the Virtual Supermarket software, for example 'The program was easy to understand', and 'The products I have purchased in the Virtual Supermarket resemble my regular food purchases' (Table 1). Answers were measured on a 5-point Likert scale. After finishing the questionnaire, all data were stored and send to our server automatically. Data were collected in Excel files and converted into SPSS Statistical Software version 15.0 (SPSS Inc., Chicago, IL, USA) for further analysis.

**Table 1:** Questionnaire on appreciation of the Virtual Supermarket (n = 66)

	Item		n
1	The program was easy to understand	Agree	55
		Neutral	7
		Disagree	4

2	The products I have purchased in the Virtual Supermarket resemble my regular food purchases	Agree	52
		Neutral	7
		Disagree	7

3	I could easily find my way around the Virtual Supermarket	Agree	48
		Neutral	11
		Disagree	7

4	The Virtual Supermarket contained sufficient product variety	Agree	37
		Neutral	14
		Disagree	15

5	There were sufficient choice options in the Virtual Supermarket	Agree	36
		Neutral	9
		Disagree	21

6	The stock of the Virtual Supermarket is a fair representation of the stock of a real supermarket	Agree	34
		Neutral	11
		Disagree	21

7	I could relatively easily find all the products in the Virtual Supermarket	Agree	26
		Neutral	19
		Disagree	21

8	I was able to imagine doing my real-life grocery shopping in the Virtual Supermarket	Agree	51
		Neutral	8
		Disagree	7

#### Results

In total, n = 66 participants completed the pilot study. Most of the sample was female (n = 49) and native Dutch (n = 60) (Table 2). Educational level was fairly evenly spread among the sample. None of the participants had major technical problems with the program and all data were accurately sent to our server. Results regarding appreciation of the application (Table 1) revealed that the majority of respondents considered the Virtual Supermarket easy to understand (*n *= 55 agree, *n *= 7 neutral, *n *= 4 disagree) and could easily find their way around the Virtual Supermarket (*n *= 48 agree, *n *= 11 neutral, *n *= 7 disagree) (Table 2). Around half of the respondents agreed that the Virtual Supermarket had a sufficient variety of products in stock (*n *= 37 agree, *n *= 14 neutral, *n *= 15 disagree), and thought that the stock of the Virtual Supermarket resembled the stock of a real supermarket (*n *= 34 agree, *n *= 11 neutral, *n *= 21 disagree). Moreover, most respondents indicated that the products they selected in the Virtual Supermarket corresponded to their normal weekly groceries (*n *= 52 agree, *n *= 7 neutral, *n *= 7 disagree). Both the results on participant level and at the technical level imply that the Virtual Supermarket is a good-quality tool to measure shopping behaviour.

**Table 2:** Participant characteristics Pilot Study (n = 66)

Variable		n
Gender	Female	49
	Male	17

Ethnicity	Dutch	60
	Other	6

Education level^1^	Low	15
	Medium	24
	High	27

Employment status	Employed	39
	Retired	11
	Other	16

Level of responsibility for groceries in real life	Totally responsible	40
	Largely responsible	10
	Shared responsibility with partner	9
	Partially responsible	4
	Not responsible	3

Weekly budget for groceries in real life (€)	0-40	6
	40-80	31
	80-120	23
	≥ 120	6

^1 ^Education level: low = primary school or lower secondary level; medium = higher secondary or intermediate vocational level; high = higher vocational or university level.

## Discussion

The Virtual Supermarket is a three-dimensional web-based software application which was designed to simulate a real supermarket. Unique features of the tool include that it enables researchers to easily modify research conditions (e.g., different pricing or labelling strategies) and in this way study different types of environmental interventions that might stimulate healthy eating without having to rely on a complex implementation process. Although the Virtual Supermarket can never replace studies in a real-life setting, it is an innovative research tool with great potential to assist in gaining further insight into consumer behaviour.

The Virtual Supermarket was pilot-tested among n = 66 consumers. This study revealed that a large majority of the participants found the program easy to understand and indicated that their virtual purchases largely corresponded to their groceries in real life. On the basis of this pilot study it can be concluded that the Virtual Supermarket is a useful tool for studying consumer food purchasing behaviour. A limitation of the pilot study was that it did not include age measurements. Age may be of importance since younger participants may find it easier to work with a computerized program than older participants. However, a growing number of people have access to a computer and computerized tools are even used in studies specifically aimed at older adults [18]. Based on this, it is not expected that the workability of the Virtual Supermarket is rated differently among different age groups. Nevertheless, during recruitment we made an effort to include participants from different age groups and focused on middle-aged people. Another limitation of the pilot study is that the number of participants may be considered low. However, we included participants with varying education levels and backgrounds and therefore believe the pilot study offers a fair impression of how the Virtual Supermarket is perceived among the general public.

### Implications

The Virtual Supermarket is a valuable tool precisely because it offers high levels of controllability, it allows prices to be easily be manipulated, and because a complex implementation in a real-life setting is avoided. Nevertheless, the feasibility of interventions in real life settings should not be neglected [15]. Behaviour in a real setting may differ from a virtual setting because real life concerns real money and real products and this may lead virtual shopping to be taken less seriously. It is therefore of major importance to validate the Virtual Supermarket against real-life food purchases. At the moment, we are planning such a validation study. As the Virtual Supermarket has not yet been validated against actual food purchases, this is a major limitation which should be considered when applying the tool for research. At the same time, however, electronic shopping is becoming increasingly common these days, and a large majority of participants in the Virtual Supermarket study indicated that their virtual groceries resembled their normal groceries. Moreover, there is evidence that peoples' virtual behavior largely corresponds with their actual behavior. Sharpe et al. (2008) validated food and beverage choices made in a virtual road trip survey by comparing those choices with choices made in a real McDonalds a week later. The authors concluded that peoples' simulated purchasing behavior is highly predictive of their real behavior [19]. Virtual shopping behaviour may thus also be fairly comparable to real-life shopping behaviour.

A second limitation of the Virtual Supermarket is the product selection procedure. Due to feasibility issues we had to decrease the usual supermarket stock of thousands of products to 512 products in the Virtual Supermarket. This was undertaken by the researchers and not based on sales data. Due to this process some popular products may be not available in the Virtual Supermarket and people were limited in the number of choices. The tool may therefore not give a complete picture of complex phenomena such as product selection within food categories and the cross price elasticity of demand [20]. Nevertheless, the application presents a representative sample of the stock of an average supermarket and contains a fair number of products from all product categories (for example, 19 types of cheese, 20 types of chocolate and 41 types of vegetables). Also, a majority of the participants indicated that the Virtual Supermarket had sufficient choice options. Furthermore, because the application has the features of a real supermarket, it may provide better quality predictions than experiments using small-scaled settings such as vending machines or laboratory settings. To our knowledge, there are no other software tools available comparable to the Virtual Supermarket. While there are some examples, they have the appearance of a web shop, where products can be selected through product lists [21]. In addition, there are examples of supermarkets in a laboratory setting, such as that of Epstein et al. [22]. A major limitation of this laboratory setting is that the number of products is very limited (around 60) and not all food categories are represented. The Virtual Supermarket is unique in that it is a three-dimensional software application in which the shopping experience closely resembles that of a real supermarket. Moreover, alongside the features described, the Virtual Supermarket can be adapted for future studies by placing other products of interest in the supermarket, that is different brands of products or products that fit the dietary patterns of other countries.

A final remark on the current version of the Virtual Supermarket concerns the fact that the application is based on a Dutch supermarket which means that it can not be used in other countries without some adaptations. However, the software is constructed in such a way that complex alterations are possible. The application base can be used to build future versions. Also, we are currently working with SARA on developing an English-language version of the Virtual Supermarket.

## Conclusions

Pricing strategies are frequently cited as a promising tool to stimulate healthier food choices. However, experimental evidence on the effectiveness of such strategies is limited. This lack is merely due to the complex nature of good-quality pricing experiments in larger food environments such as supermarkets. In order to find a solution to this problem, we designed a unique research tool: the Virtual Supermarket. The Virtual Supermarket is a three dimensional software application which was designed based on a real supermarket. A pilot study among n = 66 participants revealed that the application stores data accurately and is rated well by its users. The participants had no trouble understanding the application and reported that the purchases they made in the Virtual Supermarket were similar to their real-life purchases. Although the Virtual Supermarket can never replace studies in a real-life setting, it is an innovative research tool with a great potential to assist in gaining further insight into consumer behaviour.

### Availability and requirements

*Project name*: The Virtual Supermarket

*Project home page*: Detailed information about the Virtual Supermarket application, downloads and configuration can be found at: http://www.bioinformatics.org/supermarket/index.html.

The application can be downloaded for: Windows: http://www.falw.vu/boodschappen/Supermarket_0027_windows.zip; Mac: http://www.falw.vu/boodschappen/Supermarket_0027_mac_universal.zip. The installation consists of unpacking a compressed file to the desired location.

*Operating systems*: Windows, Mac

*Programming language*: Not applicable. The application was developed in Unity which is both a game engine and a game development environment. Changes with respect to research conditions (e.g., pricing, signage, etc.) can be made using a text editor. How this works is described at the projects' homepage

*Other requirements*: Not applicable

*Licence*: The application can be obtained for free for academic purposes without any further agreements.

*Any restrictions to use by non-academics*: The application in its current form may not be used for commercial purposes. Before using the application, non-academics should contact us via wilma.waterlander@vu.nl or ingrid.steenhuis@vu.nl.

## Competing interests

One of the authors (MS) is affiliated to SARA Computing and Networking Services Amsterdam, the company that collaborated in the development of the Virtual Supermarket. SARA is the Dutch National High Performance Computing and e-Science Support Centre and the Dutch supernode in the international Science Grid. SARA is a private company but collaborates with VU University Amsterdam on a non-profit basis.

## Authors' contributions

WEW contributed to the conception and design of the Virtual Supermarket, to the collection, processing and interpretation of the data from the pilot study and was also involved in drafting the manuscript. MS is the software programmer for the Virtual Supermarket and contributed to the conception and design of the application. DL contributed to the collection, processing and interpretation of the data from the pilot study. IHMS contributed to the conception and design of the Virtual Supermarket, the interpretation of the data from the pilot study, the acquisition of funding, and was also involved in drafting the manuscript. All authors have read and approved the final manuscript.

## Endnotes

^a ^SARA is the Dutch National High Performance Computing and e-Science Support Centre, and the Dutch supernode in the international Science Grid. SARA collaborates with the VU University Amsterdam on a non-profit base.

## Pre-publication history

The pre-publication history for this paper can be accessed here:

http://www.biomedcentral.com/1471-2458/11/589/prepub
